# Profiling the peripheral blood T cell receptor repertoires of gastric cancer patients

**DOI:** 10.3389/fimmu.2022.848113

**Published:** 2022-07-28

**Authors:** Mengyao Wang, Peng Gao, Laifeng Ren, Jingjing Duan, Silu Yang, Haina Wang, Hongxia Wang, Junning Sun, Xiaoyan Gao, Bo Li, Shuaicheng Li, Wen Su

**Affiliations:** ^1^ Department of Computer Science, City University of Hong Kong Shenzhen Research Institute, Shenzhen, China; ^2^ BGI-Shenzhen, Shenzhen, China; ^3^ Department of Immunology, Shanxi Province Cancer Hospital/ Shanxi Hospital Affiliated to Cancer Hospital, Chinese Academy of Medical Sciences/Cancer Hospital Affiliated to Shanxi Medical University, Taiyuan, China

**Keywords:** TCR repertoire, next-generation sequencing, gastric cancer, biomarker, antitumor immunity

## Abstract

Cancer driven by somatic mutations may express neoantigens that can trigger T-cell immune responses. Since T-cell receptor (TCR) repertoires play critical roles in anti-tumor immune responses for oncology, next-generation sequencing (NGS) was used to profile the hypervariable complementarity-determining region 3 (CDR3) of the TCR-beta chain in peripheral blood samples from 68 gastric cancer patients and 49 healthy controls. We found that most hyper-expanded CDR3 are individual-specific, and the gene usage of TRBV3-1 is more frequent in the tumor group regardless of tumor stage than in the healthy control group. We identified 374 hyper-expanded tumor-specific CDR3, which may play a vital role in anti-tumor immune responses. The patients with stage IV gastric cancer have higher EBV-specific CDR3 abundance than the control. In conclusion, analysis of the peripheral blood TCR repertoires may provide the biomarker for gastric cancer prognosis and guide future immunotherapy.

## Introduction

Gastric cancer (GC) is one of the most common and lethal malignancies worldwide, and ranks as the second leading cause of cancer-related deaths (1). Due to the lack of specific symptoms and effective early diagnostic methods, most patients with GC are diagnosed at an advanced stage with local or distant metastases and have a poor prognosis (2). Therefore, to improve the prognosis of patients with GC, it is essential to search for novel molecular markers that can help in early diagnosis, risk assessment, and therapy.

T cells play important roles in anti-tumor immunity through the specific recognition and combination of tumor antigens–MHC complex by T-cell receptors (3). T cell-based cancer immunotherapies represent a new era in cancer therapy, such as chimeric antigen receptor (CAR)-engineered T cells and immune checkpoint blockade (4, 5). TCR is mainly composed of α and β chains and is expressed on cell surfaces. To recognize a large variety of antigens, TCR gene recombination, a process of somatic rearrangement of variable (V), diversity (D), and joining (J) genes, generates a diverse set of TCRs (6). TCRs are extremely specific and diverse, which are predominantly determined by the amino acid sequences of the hypervariable complementarity-determining region 3 (CDR3) by random rearrangement and junction region mutation of V(D) J regions (7, 8). The total number of potential expressed rearranged TCR (mainly CDR3) sequences in an individual is referred to as the TCR repertoire, and the diversity of TCR repertoire gives it the ability to bind a wide variety of antigens (9). Therefore, detecting the diversity of the TCR repertoire mainly based on variation in the CDR3 region in each TCR Vβ family could reflect the diversity of cellular immunity (10). Latest methodological advancements, especially next-generation sequencing (NGS) technology, have allowed rapid and accurate identification and quantification of the TCR repertoire (11–13).

Profiling of TCR repertoire will be useful not only in understanding the potential immunological mechanisms of tumorigenesis but also in the exploration of new tumor diagnosis, treatment, and prognostic indicators. Several recent studies have shown that the diversity of TCR repertoire is associated with the prognosis of patients with cancer, such as breast cancer (14), liver cancer (15), lung cancer (16), and GC (17). However, to date, studies on TCR repertoire determined by NGS in GC are limited. Kuang et al. (17) found the heterogeneity of tissue-infiltrating TCR repertoire during carcinogenesis. Peripheral blood is a noninvasive and readily available specimen source, and studies have suggested that the peripheral repertoire is altered in patients with cancer and may reflect aspects of the disease (10). Therefore, in this study, high-throughput sequencing was used to systematically study the CDR3 diversity of TCR β chains in blood from GC patients and healthy controls to characterize the TCR diversity associated with GC and to explore new strategies and targets for the diagnosis, treatment, and prognosis of GC.

## Method

### Sample collection

The peripheral blood samples of patients diagnosed with GC admitted to Shanxi Cancer Hospital from 2020 to 2021 and healthy people were collected. Inclusion criteria include (1) patients who were diagnosed with GC for the first time on admission, and (2) patients without other tumors and without any treatments. The inclusion criteria of the control population include (1) the physical examination population of our hospital who were similar in age to GC patients, and (2) those who had no important potential diseases. As experiments involve human materials, all sample requests, collection, and processing are informed and agreed in writing by patients and in accordance with the ethical guidelines and norms of academic institutions. This study was approved by the Ethics Committee of Shanxi Province Cancer Hospital.

The peripheral blood of 68 cancer patients was collected for TCR beta-chain repertoire sequencing, and the peripheral blood from 49 healthy subjects was obtained as controls (**Supplementary Table 1**). The blood sample of each sample was divided into 2 ml of peripheral anticoagulant blood and 2 ml of non-anticoagulant blood. One milliliter of non-anticoagulant blood was used for DNA extraction, and the rest was used for cell surface molecular detection. Serum was separated by centrifugation from the anticoagulant blood and stored at −80°C for later use. All the above operations were completed within 24 h.

### Library preparation and sequencing

Genome DNA was extracted from 1 ml of non-anticoagulant peripheral blood using the DNeasy Blood & Tissue Kit (QIAGEN,166039685) according to the manufacturer’s protocol. The concentration and purity of extracted DNA were determined by Nanodrop 2000 and stored at −80°C for later use. For each sample, 200–500 ng of DNA was used for library preparation and sequencing. The first multiplex PCR goes 10 cycles. Multiplex PCR primers include 30 TRB-V primers and 13 TRB-J primers in QIAGEN Multiplex PCR Master Mix, target the conserved regions of TRBV and TRBJ genes, and were designed to specifically amplify the complementarity-determining regions—CDR3. The second simple PCR utilizes a universal primer and goes 25 cycles, which is attached with the 3’ adapter “AAGTCGGAGGCCAAGCGGTCTTAGGAAGACAA” and the 5’ adapter “AAGTCGGATCGTAGCCATGTCGTTCTGTGAGCCAAGGAGTTG” to generate sequencing libraries, and then cooled to 4°C. The TCR beta-chain sequencing libraries were constructed using the Phusion High-Fidelity PCR Master Mix with HF Buffer (NEB) according to the instructions, and then were sequenced on the BGISEQ-500 platform with 100-bp paired-end reads (18).

Exome library construction was performed with 400 ng of DNA of each sample; the library was enriched by array hybridization (MGIEasy Exome Capture V4 Probe Set) and then sequenced on the MGISEQ-2000 platform with 100-bp paired-end reads, according to the manufacturer’s standard procedure.

### Detection of cell surface molecules by flow cytometry

Flow cytometry was used to determine the percentage and absolute number of lymphocyte subtypes in whole blood samples by using a six-color fluorescent monoclonal antibody kit combined with an absolute count microsphere kit (QuantoBio, Beijing, China). Briefly, peripheral blood cells were stained with the six-color fluorescent monoclonal antibody including PerCP-Cy5.5-anti-CD45, FITC-anti-CD3, PC7-anti-CD4, APC-Cy7-anti-CD8, APC-anti-CD19, and PE-anti-CD16+56+ according to the manufacturer’s protocol. After that, the samples were treated with hemolysin and lysed erythrocytes. Before flow cytometry detection, absolute counting microspheres of the same volume as the test sample were added and thoroughly mixed. Then, the cells were analyzed by the BD LSRFortessa cell analyzer (Becton-Dickinson FACSCanto II), and the percentage and absolute number of lymphocyte subtypes were analyzed by CellQuest Pro software (Becton-Dickinson FACSCanto 3.1).

### EBV EA-IgG detection

Commercial EBV EA-IgG ELISA kit (Autobio, Zhengzhou, China) was used to detect EBV EA-IgG in serum, strictly according to the instructions.

### Bioinformatics analysis

The raw sequencing data were filtered with SOAPnuke (19) (v1.5.3) to remove reads with adapter contamination, reads with more than 10% N bases, and low-quality reads containing more than 50% low-quality bases (base quality value < 15, **Supplementary Table 2**). After the data filtration, the clean sequencing data were aligned to the human TRBV, TRBD, and TRBJ reference database using MiXCR (20)(v3.0.13, **Supplementary Table 3**) and then mapped reads were assembled into clonotypes, which refer to the unique TRBV–TRBJ CDR3 nucleotide sequence. The MiXCR applied a heuristic multilayer clustering method to correct the PCR and sequencing error. The TCR clonotypes with clone count and fraction were exported for further analysis. The R package “immunarch” (21) (v0.6.6) was employed to perform repertoire analysis, such as the relative abundance of CDR3, TRBV/TRBJ gene usage, estimation of repertoire diversity, and antigen-specific annotation of CDR3. The statistical analysis was performed using R software (v3.6.0), and the Wilcoxon test was adopted to compare the difference between the two groups. The whole-exome sequencing (WES) data were filtered using SOAPnuke with default settings and then HLA typing was performed with OptiType (22) (v1.3.1). We used the Fisher test to evaluate the enrichment of each HLA allele in the tumor group and control group.

## Results

### Clonal expansion in healthy controls and cancers

On average, 9.27 × 10^6^ TCR-β sequences were obtained from each individual (7.71 × 10^6^ in the healthy control group and 1.04 × 10^7^ in the tumor group). The mean number of unique CDR3 of the healthy and the tumor group is 11,655.4 and 12,875.8, respectively (**Supplementary Figure 1C**). Also, we found that there is no significant difference in the absolute number of CD3^+^ cells between the tumor and control group (Wilcoxon test, *p* = 0.629). The length of CDR3 in both healthy controls and cancer patients showed a normal distribution (**Supplementary Figure 1A**). We defined the CDR3 with a frequency of >1% to be hyper-expanded CDR3, the CDR3 with a frequency of >0.1% to be large CDR3, the CDR3 with a frequency of >0.01% to be medium CDR3, and the CDR3 with a frequency of ≤0.01% to be small CDR3 (23). The proportion of both large clones and highly expanded clones was comparable between the healthy controls and the cancer group (**Supplementary Figure 1D**).

**Figure 1:**
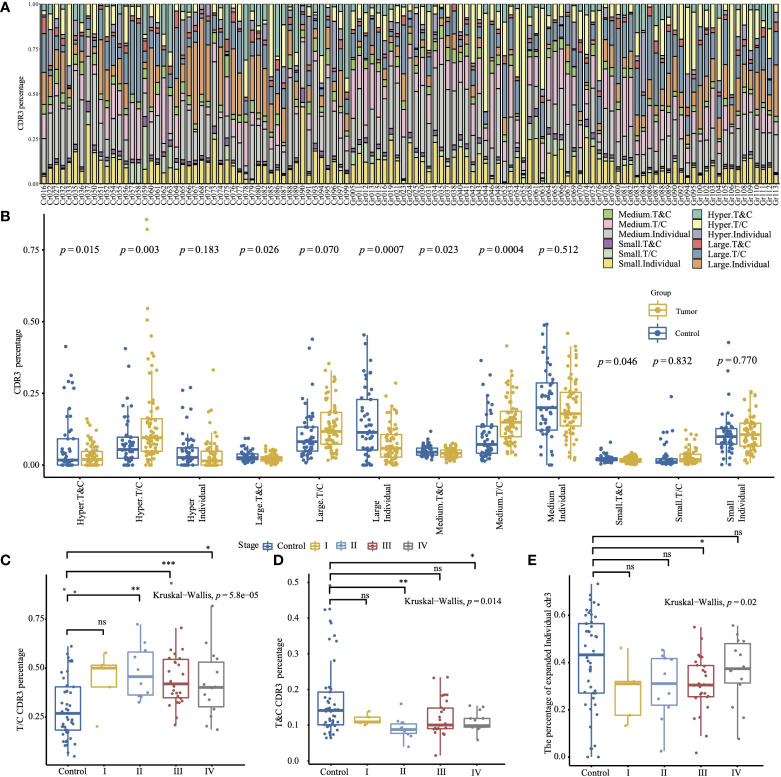
The relative abundance with specific frequencies of the different CDR3 groups is based on the shared CDR3 between the tumor group and the healthy control group. **(A)** The CDR3 percentage of different groups in all samples. **(B)** The comparison of the CDR3 percentage of different groups between the tumor and the control group (two-sided *t*-test). **(C)** Comparison of T/C CDR3 percentage between the control group and sub-tumor group of different tumor stages. **(D)** Comparison of T&C CDR3 percentage between the control group and sub-tumor group of different tumor stages. **(E)** Comparison of expanded individual CDR3 percentage between the control group and sub-tumor group of different tumor stages. (The convention for symbols indicating statistical significance, ns: *p* > 0.05; *p ≤ 0.05; **p ≤ 0.01; ***p ≤ 0.001. The two-sided Wilcox test was used for two-group comparison, and the Kruskal–Wallis test was used for comparing more groups.).

We further divided TCR CDR3 into three groups (“T&C”, “T/C”, and “Individual”) based on the shared CDR3 between the healthy control group and the tumor group. The CDR3 in the “T&C” group is shared by both the healthy control group and the tumor group (shared by at least one control sample and at least one tumor sample), the “T/C” group involved the CDR3 shared by the tumor group or the control group (if the CDR is identified in at least two control samples, but does not exist in the tumor samples, or identified in at least two tumor samples, but does not exist in the control samples), and the “Individual” group contains the unique CDR3 of each individual; 13.6% of CDR3 are shared by the tumor group and the control group, 32.0% are shared by the control group, 49.2% are shared by the tumor group, and an average of 46.1% of CDR3 are unique for each individual (**Figure 1A**; **Supplementary Table 4**). Most hyper-expanded CDR3 are individual-specific in both the tumor group and the healthy control group. The control group has a significantly higher percentage of the small, medium, large, and hyper-expanded CDR3 shared by both the tumor group and the control group (*p* = 0.046, *p* = 0.023, *p* = 0.026, *p* = 0.015), and these CDR3 display different distributions *via* different stages (*p* = 0.014, Kruskal–Wallis test, **Figure 1D**). The large individual-specific CDR3 of the tumor group accounts for a significantly higher proportion than the healthy control group (*p* = 0.0007, **Figure 1B**). CDR3 specifically shared by the tumor group occupied a higher proportion than CDR3 shared by the control group, and shows different distribution *via* different stages (*p* = 5.8e-5, Kruskal–Wallis test, **Figure 1C**). The distribution of the expanded (medium, large, and hyper) individual-specific CDR3 has a significant difference (*p* = 0.02, Kruskal–Wallis test, **Figure 1E**).

### TRBV and TRBJ gene usage comparison between the healthy controls and cancer patients

**Figure 2:**
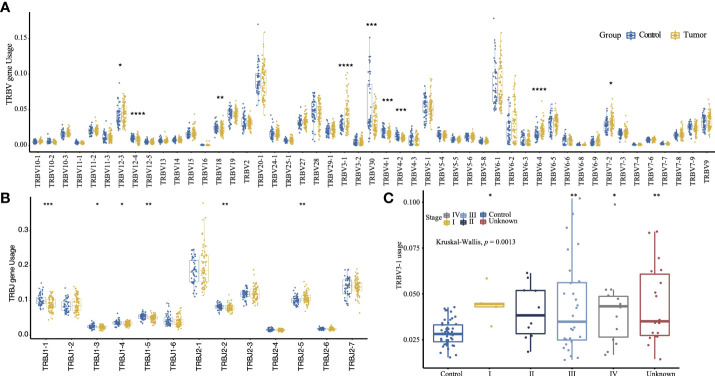
The TRBV **(A)** and TRBJ **(B)** usage comparison between the tumor group and control group (two-sided *t*-test). **(C)** Comparison of *TRBV3-1* gene usage between the control group and the sub-tumor group of different tumor stage. (The convention for symbols indicating statistical significance, ns: *p* > 0.05; *p ≤ 0.05; **p ≤ 0.01; ***p ≤ 0.001; ****p ≤ 0.0001; Kruskal–Wallis test.).

The gene usage of TRBV and TRBJ was determined by the clonotypes assembled by MiXCR. *TRBV20-1* (9.24%) and *TRBV6-1* (8.42%) are the most frequently used TRBV genes, and *TRBJ2-1* (19.2%) and *TRBJ2-7* (13.9%) are the most frequently used TRBJ genes. The tumor group has a significantly lower gene usage of *TRBV30*, *TRBV12-4*, *TRBV4-1*, *TRBV4-2*, *TRBV18*, *TRBJ1-1*, *TRBJ1-5*, and *TRBJ2-2*. The *TRBV3-1*, *TRBV6-4*, *TRBV12-3*, *TRBV7-2*, and *TRBJ2-5* are more frequently used in tumor groups (**Figures 2A**, **B**). Moreover, the tumor group has a higher *TRBV3-1* gene usage frequency than the control group regardless of tumor stage (*p* = 0.0013, Kruskal–Wallis test, **Figure 2C**). As only A*02:01 is significantly enriched in the tumor group (**Supplementary Table 5**), we compared the gene usage of TRBV and TRBJ between samples with or without A*02:01 to identify the TRBV or TRBJ gene usage bias, only *TRBJ2-3* has a significant relationship with A*02:01, but it has no difference between the tumor group and the control group (**Supplementary Figure 2**). We also compared the TRBV–TRBJ combination usage, and the tumor group and control group differ greatly. The highest V–J pairing usage in the tumor group is *TRBV6-1–TRBJ1-6*, while *TRBV30–TRBJ2-2* is the most frequently used V–J pair in the control group (**Supplementary Figure 3**). The combined usage of *TRBV12-3–TRBJ2-2*, *TRBV12-3–TRBJ2-5*, *TRBV4-1–TRBJ1-1*, and *TRBV15–TRBJ1-2* is significantly associated with tumor stage (**Supplementary Figure 4**, Kruskal–Wallis test, *p* = 2.8e-05, *p* = 0.00042, *p* = 4.8e-06, *p* = 0.00038).

### Identification of hyper-expanded tumor-specific TCR CDR3

**Figure 3:**
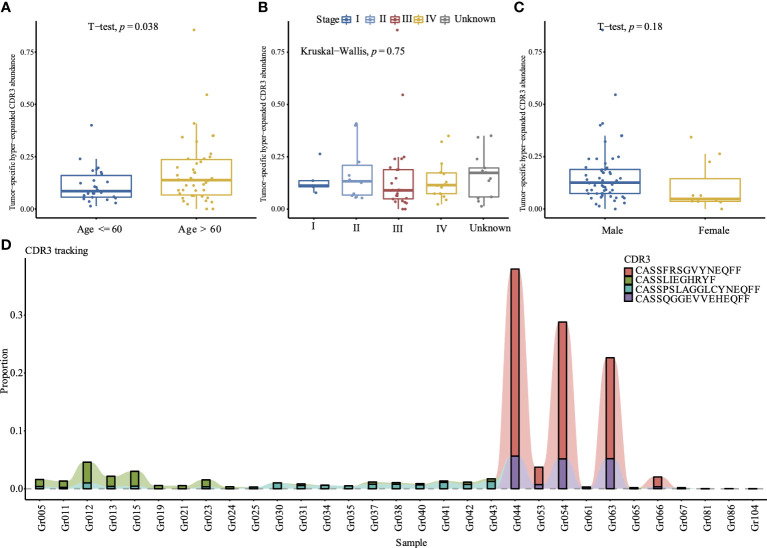
Comparison of the tumor-specific hyper-expanded CDR3 abundance between different age **(A)**, tumor stages **(B)**, and gender **(C)** in 68 tumor samples. (D) Tracking of top 4 hyper-expanded tumor specific TCR CDR3. The y-axis is the proportion of each CDR3 among the repertoire, samples without these 4 CDR3 are excluded.

A total of 1,175,884 unique CDR3 amino acids were identified from the 117 individuals. Among them, 461,554 CDR3 were found in healthy controls, 38,922 CDR3 were shared between healthy controls and cancer patients, 675,408 CDR3 were found in cancer patients, and 63,712 CDR3 were generated by at least two cancer patients; among these CDR3, we identified 374 hyper-expanded tumor-specific CDR3. After removing CDR3 that were recorded by the ImmuneCODE™ database (24), 292 CDR3 were retained to calculate the abundance of tumor-specific hyper-expanded CDR3, which is significantly higher in the elder group (**Figure 3A**), but comparable in different tumor stage (**Figure 3B**) and gender (**Figure 3C**). The tumor-specific CDR3 “CASSFRSGVYNEQFF” is hyper-expanded across Gr044, Gr053, Gr054, Gr063, and Gr066 (**Figure 3D**); the CDR3 cluster shared antigen specificity with the CDR3 “CASSFRSGVYNEQFF” is widespread among the tumor group (52/68), this CDR3 cluster contains 103 CDR3, the sequence logo of this cluster showed that positions 6–9 are diverse, and the first four amino acids and the last five amino acids are conserved (**Supplementary Figure 5**). “CASSQGGEVVEHEQFF” is enriched in Gr044, Gr054, and Gr063. The frequency of CDR3 “CASSLIEGHRYF” is high across Gr005, Gr011, Gr012, Gr013, Gr015, and Gr023. The CDR3 “CASSPSLAGGLCYNEQFF” was identified in 21 tumor patients, and hyper-expanded across Gr012, Gr030, Gr041, and Gr043. These hyper-expanded tumor-specific TCR CDR3 may be important for cancer immunotherapy.

### Analysis of TCR CDR3 with EBV antigen specificity

**Figure 4:**
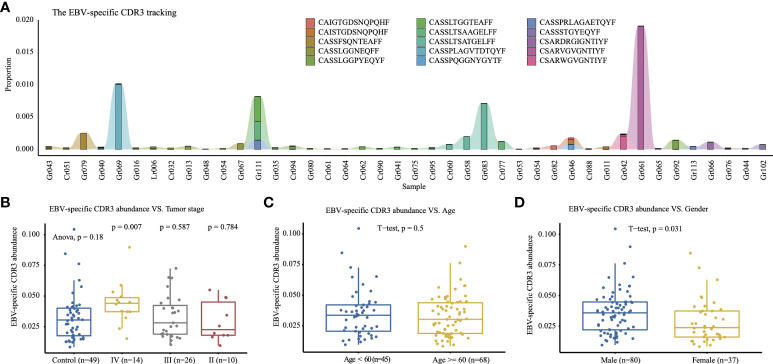
Top 15 EBV-specific CDR3. **(A)** Tracking the top 15 high-frequency EBV-specific CDR3 that have higher abundance in the tumor group; samples with a total count of EBV-specific CDR3 of less than 2,000 are excluded. The *y*-axis is the proportion of each CDR3 among the repertoire. The EBV-specific CDR3 abundance comparison of tumor stages **(B)**, age **(C)**, and gender **(D)** among 117 samples.

The TCR CDR3 were annotated with antigen-specific immune receptor databases VDJdb (25), McPAS-TCR (26), and PIRD TBAdb (27), and 688 EBV (Epstein–Barr Virus)-specific TCR CDR3 were identified, including the hyper-expanded CDR3 “CSARVGVGNTIYF” in Gr061 (1.9%), the hyper-expanded “CASSPLAGVTDTQYF” in Gr069 (1.0%), and the large CDR3 “CASSLTSATGELFF” in Gr083 (0.7%) (**Figure 4A**). As the limitation of incomplete antigen-specific immune receptor databases, we clustered the CDR3 amino acid sequences with iSMART (28), which aggregated T-cell receptor CDR3 sequences into antigen-specific clusters based on the pairwise local CDR3 alignment scores. The CDR3 clusters containing EBV-specific CDR3 were identified as EBV-specific CDR3 clusters. We defined the EBV-specific CDR3 abundance as the frequency of all CDR3 in the EBV-specific CDR3 clusters. As Camago et al. have reported that male patients under the age of 60 have a higher incidence of EBV-positive GC (29), we compared the EBV-specific CDR3 abundance in age, gender, tumor stage, and other risk factors. The male group has a significantly higher EBV-specific CDR3 abundance than the female group (*t*-test, *p* = 0.031, **Figure 4D**), and the elders (age ≥; 60) tend to have lower EBV-specific CDR3 abundance (**Figure 4C**). The stage IV GC patients have a significantly higher EBV-specific CDR3 abundance than the healthy control group (*t*-test, *p* = 0.007, **Figure 4B**), which indicated that the high level EBV-specific CDR3 is a potential biomarker in cancer monitoring or diagnosis, although the EBV-positive GC was not significantly associated with tumor stage in the meta-analysis of 5,081 GC (30).

## Discussion

T cells are an important weapon of the body’s anti-tumor immune response, and they specifically recognize tumor cells through their TCR and thereby selectively remove them. The diversity of TCR enables T cells to recognize a wide variety of tumor antigens, thereby stimulating an effective anti-tumor immune response (31). Therefore, analysis of T cell-specific CDR3 sequences by high-throughput sequencing technology and bioinformatics allows us to understand the specificity and diversity of antigen receptors required in adaptive immunity, and provides more accurate TCR profiles (31). The results of TCR profiles have a great potential to make significant contributions in the clinic *via* novel approaches to diagnosis, prognosis, therapeutic decision-making, and the development of new therapeutic targets (10), such as diagnosis of tiny residual disease, monitoring the recurrence of malignant lymphoid tumors (32, 33), predicting the beneficiaries and efficacy of immune checkpoint suppressive therapy (34), and screening for tumor antigens from tumor-specific TCR clones (35).

So far, studies on the tumor-associated TCR library mainly focused on tumor-infiltrating lymphocytes. Patients with advanced cancer who cannot have surgery or biopsy for a variety of reasons may not be able to obtain tumor tissue, which may be unavailable throughout the treatment period. For these patients, it is necessary to explore an alternative to tissue biopsy that is repeatable and minimally invasive and can monitor clinical response and disease evolution in real time. Considering that T cells shuttle back and forth between tumor and systemic circulation, peripheral blood TCR bank can also reflect the anti-tumor immune status of tumor patients to a certain extent (16). Peripheral blood TCR bank analysis has gradually attracted attention recently for its minimally invasive, easy-to-obtain, dynamic analysis (16, 35). To the best of our knowledge, there have been no studies on high-throughput sequencing-based TCR library analysis in peripheral blood of GC patients. Therefore, in this study, the diversity and clone types of TCRVβ chains in peripheral blood of 68 GC patients and 49 healthy controls were analyzed, and the feasibility of TCRVβ chains as biomarkers for tumor diagnosis and treatment was discussed.

At present, conclusions on the relationship between the number of T-cell clones and tumor diagnosis and prognosis vary with different tumors and even different studies. A higher diversity was found in peripheral blood samples of the healthy population than of patients with cervical cancer (36) and lung cancer (16); however, Liu et al. found that TCR diversity of cancerous tissues was also considerably higher than that in healthy lung tissues (37). Sequencing analysis of tumor-infiltrating T cells’ CDR3 from patients with GC showed that the degree of variation in the TCR repertoire gradually increased during tumorigenesis (17). In this study, we found that although there was no significant difference in the number of unique clones between GC patients and the healthy control population, there was a significant difference in the distribution of specific clones between GC patients and the control group. These results reflect the different T-cell immune response states of some tumor patients and have potential diagnostic value of tumor.

We characterized the baseline TCR-β repertoire and found that most hyper-expanded CDR3 are individual specific, which reflect the specific selection of the VDJ recombination under the individual specific immune response. The somatic recombination of the VDJ gene segments is the basis for the generation of the diversified TCR repertoire. In our study, the *TRBV3-1* gene was enriched in the tumor group. One possible explanation for the potential diagnostic biomarker of the *TRBV3-1* gene is that the mature T cells containing this gene may be activated and expanded by tumor-associated antigens. The EBV-positive GC is an important subtype of tumor with unique genomic features and good prognosis. Both EBV-positive GC and the high EBV-specific CDR3 abundance tend to occur more in male patients; thus, the analysis of TCR repertoire data may be a good method to identify the EBV-positive GC.

Although our study has some limitations, such as the insufficient sample size, which could limit the recapitulation of our findings and the collection of an external validation set, our findings indicate that characterization of the minimally invasive blood TCR repertoire provides valuable clinical diagnosis features. Moreover, paired blood and tumor tissue samples should be analyzed and compared in subsequent extensive cohorts to accurately identify tumor specific biomarkers. Nevertheless, characterization of the peripheral blood T-cell repertoire by sequencing the CDR3 region of TCR-β chain is feasible and effective in cancer research.

In conclusion, we show that the peripheral blood TCR repertoire of patients with GC is significantly different from that of healthy individuals. The peripheral blood TCR repertoire is an abundant source to identify tumor-specific biomarkers. Furthermore, changes in the TCR repertoire during anticancer treatment may be a useful prognostic indicator and biomarker for future immunotherapy.

## Data availability statement

The datasets presented in this study can be found in online repositories. The names of the repository/repositories and accession number(s) can be found below: https://ngdc.cncb.ac.cn/omix, OMIX001106.

## Ethics statement

The studies involving human participants were reviewed and approved by Medical Ethics Committee of the Shanxi Cancer Hospital. The patients/participants provided their written informed consent to participate in this study.

## Author contributions

WS and SL designed the study. LR, JD, SY, HNW, HXW, JS, XG, and BL collected the samples and performed the experiments. MW and PG analyzed the data supervised by SL. MW, PG, and LR wrote the manuscript. All authors contributed to the article and approved the submitted version.

## Funding

This work is supported by the Key Research and Development (R&D) Projects of Shanxi Province (201903D321027 and 201703D321013), the Construction Project of Technique Development Laboratory for Cancer Immunity of Shanxi Province, and the Construction Project of Tumor Immunology Innovation Team of Shanxi Cancer Hospital.

## Acknowledgments

We thank the MGI Tech Co., Ltd. and the China National GeneBank (CNGB) for the whole-exon sequencing.

## Conflict of interest

Authors PG, XG and BL employed by company BGI-Shenzhen.

The remaining authors declare that the research was conducted in the absence of any commercial or financial relationships that could be construed as a potential conflict of interest.

## Publisher’s note

All claims expressed in this article are solely those of the authors and do not necessarily represent those of their affiliated organizations, or those of the publisher, the editors and the reviewers. Any product that may be evaluated in this article, or claim that may be made by its manufacturer, is not guaranteed or endorsed by the publisher.

## Supplementary material

Supplementary Figure 1 Relative abundance of the different CDR3 groups with different frequency. **(A)** CDR3 length distribution of the tumor and control group. **(B)** Rarefaction curves of TCR-β diversities for each sample. The tumor samples are marked in orange and the control samples in green. Solid and dashed lines denote the interpolated and extrapolated regions, the points on each curve mark the exact sample size and diversity, the shaded areas means 95% confidence intervals. **(C)** The number of unique CDR3 of the tumor and control group. **(D)** Comparison of the relative abundance CDR3 groups between the tumor and control group. **(E)** The percentage barplot of the repertoire occupied by CDR3 of a given size.

Supplementary Figure 2 The TRBV **(A)** and TRBJ **(C)** usage comparison in tumor group between samples with or without A*02:01; The TRBV **(B)** and TRBJ **(D)** usage comparison in control group between samples with or without A*02:01 (two-sided t-test).

Supplementary Figure 3 3D-barplot of the VJ combination usage frequency in tumor group **(A)** and control group **(B)**.

Supplementary Figure 4 Comparison of TRBV12.3-TRBJ2.2 **(A)**, TRBV12.3-TRBJ2.5 **(B)**, TRBV4.1-TRBJ1.1 **(C)** and TRBV15-TRBJ1.2 **(D)** combination usage between the control group and sub-tumor group of different tumor stage (Kruskal-Wallis test).

Supplementary Figure 5 The clusters plot of the top 4 hyper-expanded tumor specific TCR CDR3. Each cycle denotes a CDR3, the size of the cycle is in proportion to the frequency of the CDR3. The CDR3 shared by multiple sample are exhibited in pie chart with multiple colors. The linked CDR3 differ in just have one amino acid. The sequence logo of each CDR3 clusters are added to the cluster plot to reflect CDR3 sequence conservation.
