# Supplementary figures and images for: Profiling the peripheral blood T cell receptor repertoires of gastric cancer patients
Source: Front Immunol. 2022 Jul 28;13:848113. doi: 10.3389/fimmu.2022.848113 (PMC9367216; doi:10.3389/fimmu.2022.848113)

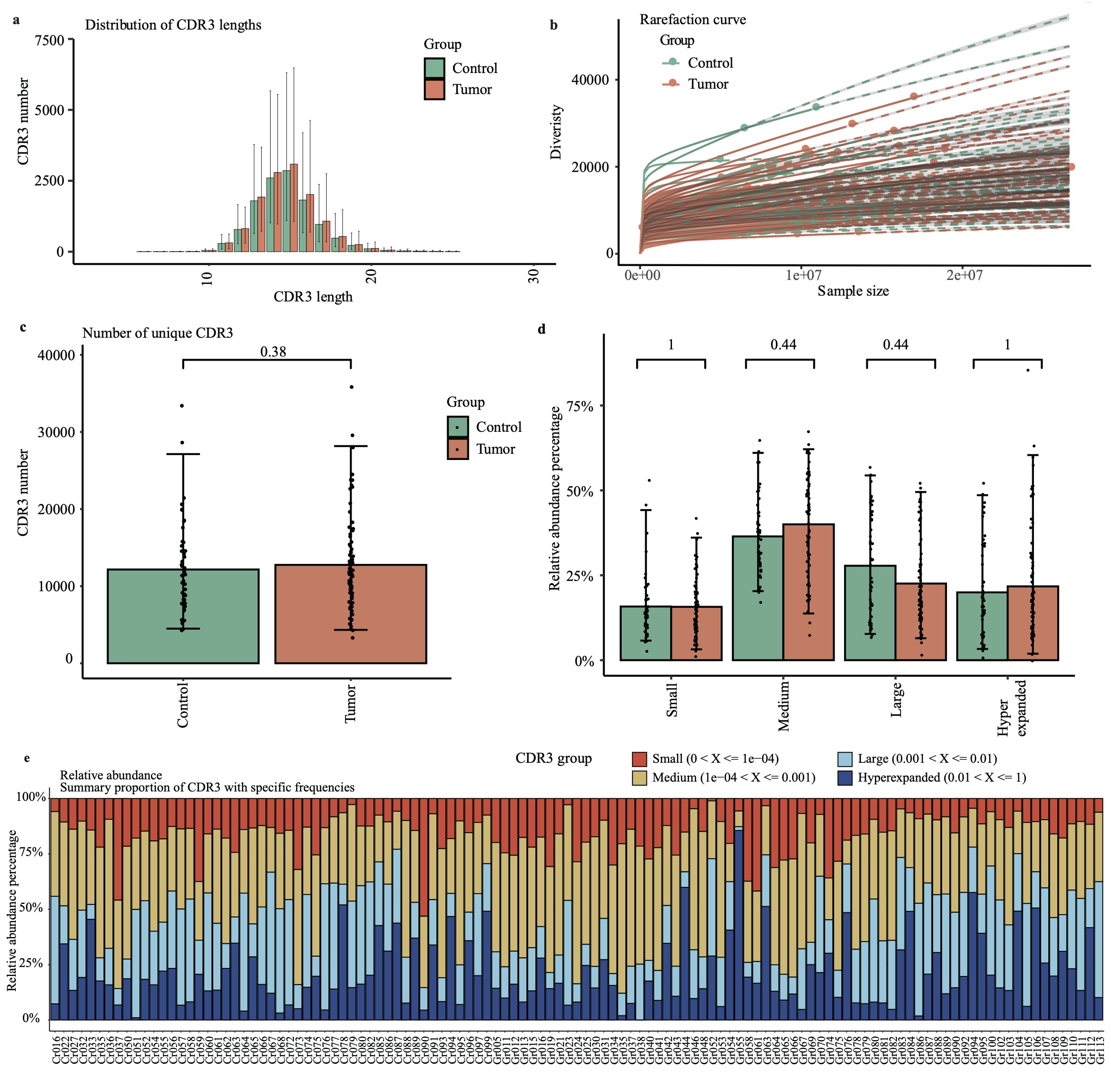

Supplement: Supplementary Figure 1 — Relative abundance of the different CDR3 groups with different frequency. (A) CDR3 length distribution of the tumor and control group. (B) Rarefaction curves of TCR-β diversities for each sample. The tumor samples are marked in orange and the control samples in green. Solid and dashed lines denote the interpolated and extrapolated regions, the points on each curve mark the exact sample size and diversity, the shaded areas means 95% confidence intervals. (C) The number of unique CDR3 of the tumor and control group. (D) Comparison of the relative abundance CDR3 groups between the tumor and control group. (E) The percentage barplot of the repertoire occupied by CDR3 of a given size. [file Image_1.jpeg]

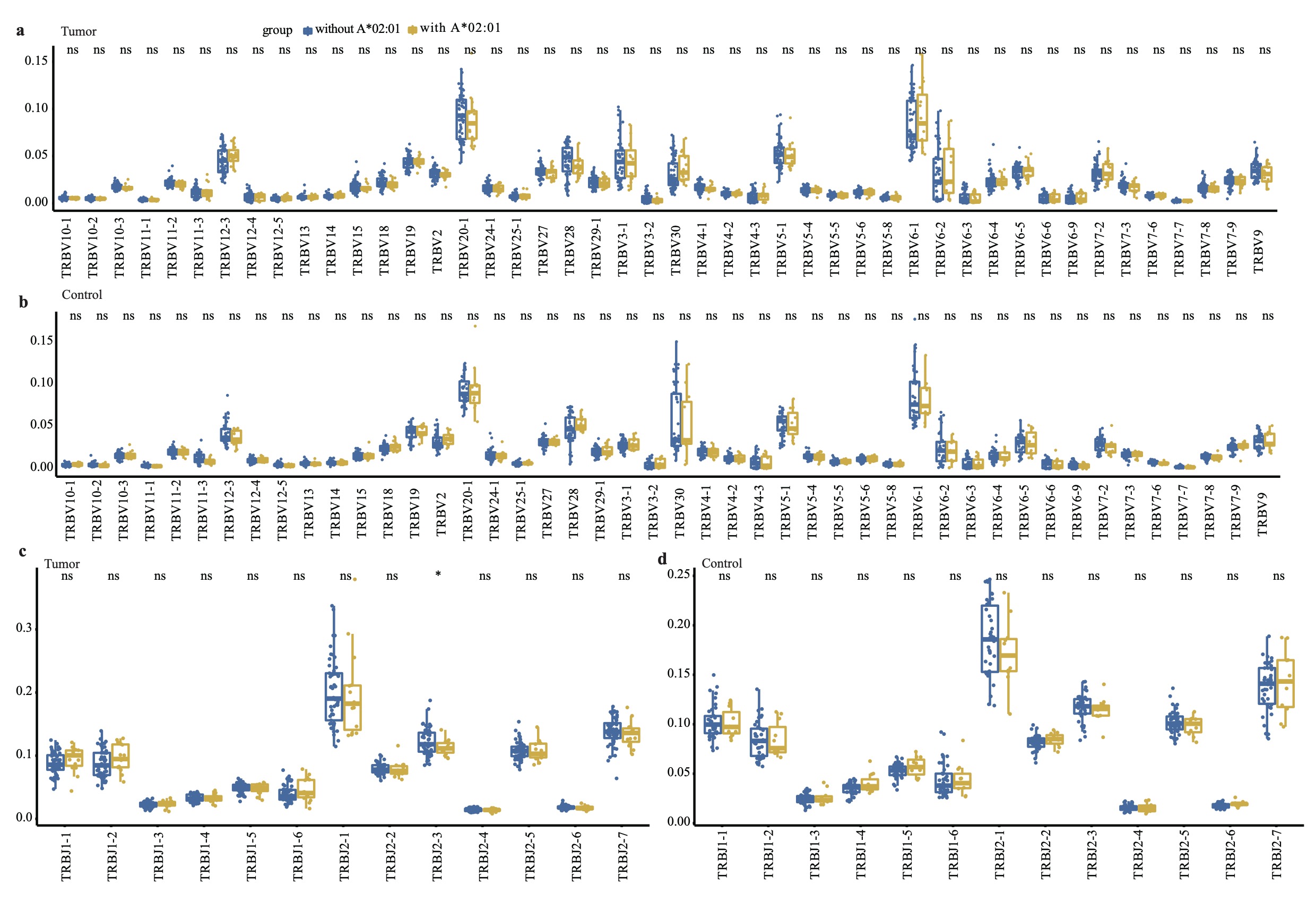

Supplement: Supplementary Figure 2 — The TRBV (A) and TRBJ (C) usage comparison in tumor group between samples with or without A*02:01; The TRBV (B) and TRBJ (D) usage comparison in control group between samples with or without A*02:01 (two-sided t-test). [file Image_2.jpeg]

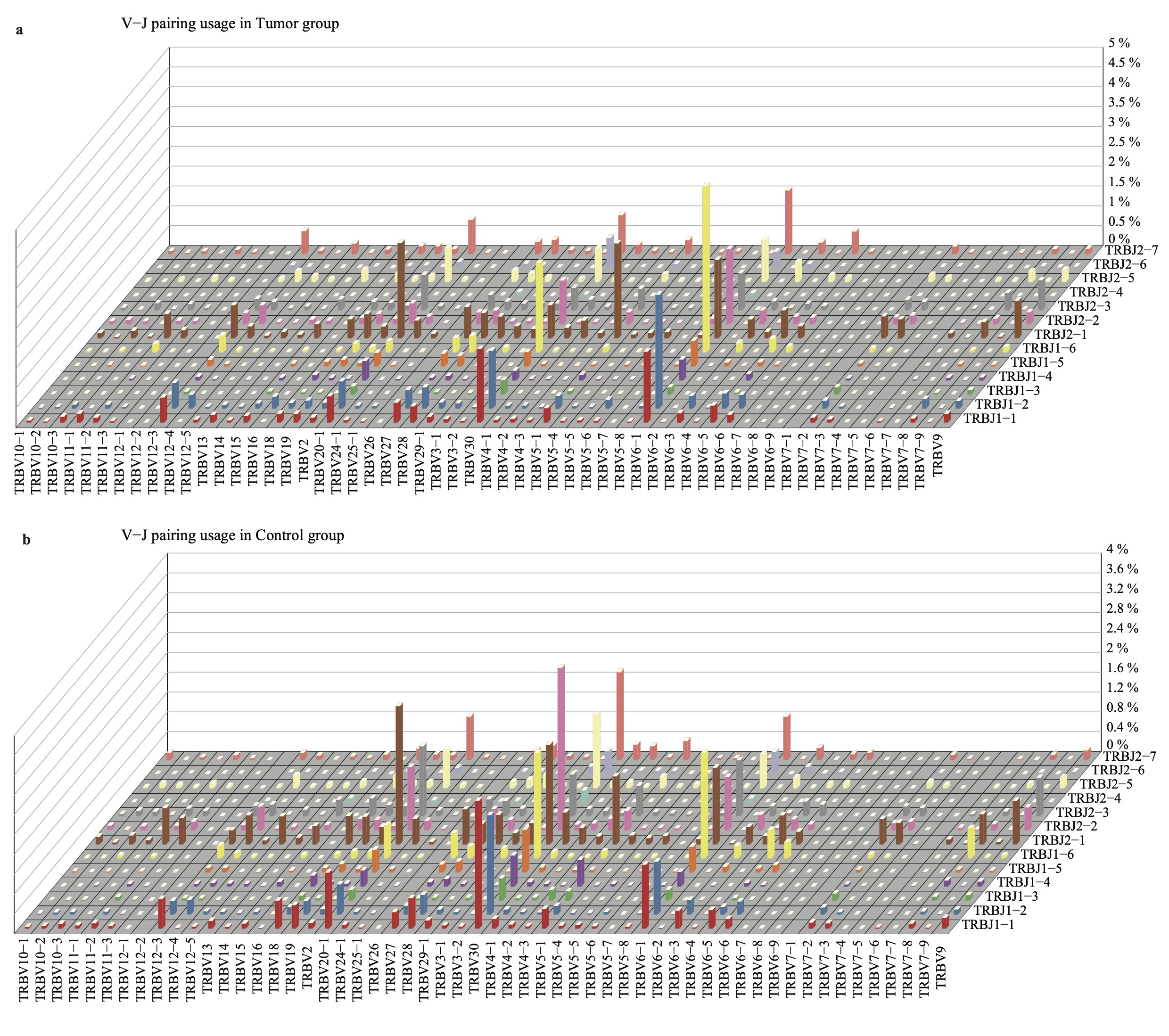

Supplement: Supplementary Figure 3 — 3D-barplot of the VJ combination usage frequency in tumor group (A) and control group (B). [file Image_3.jpeg]

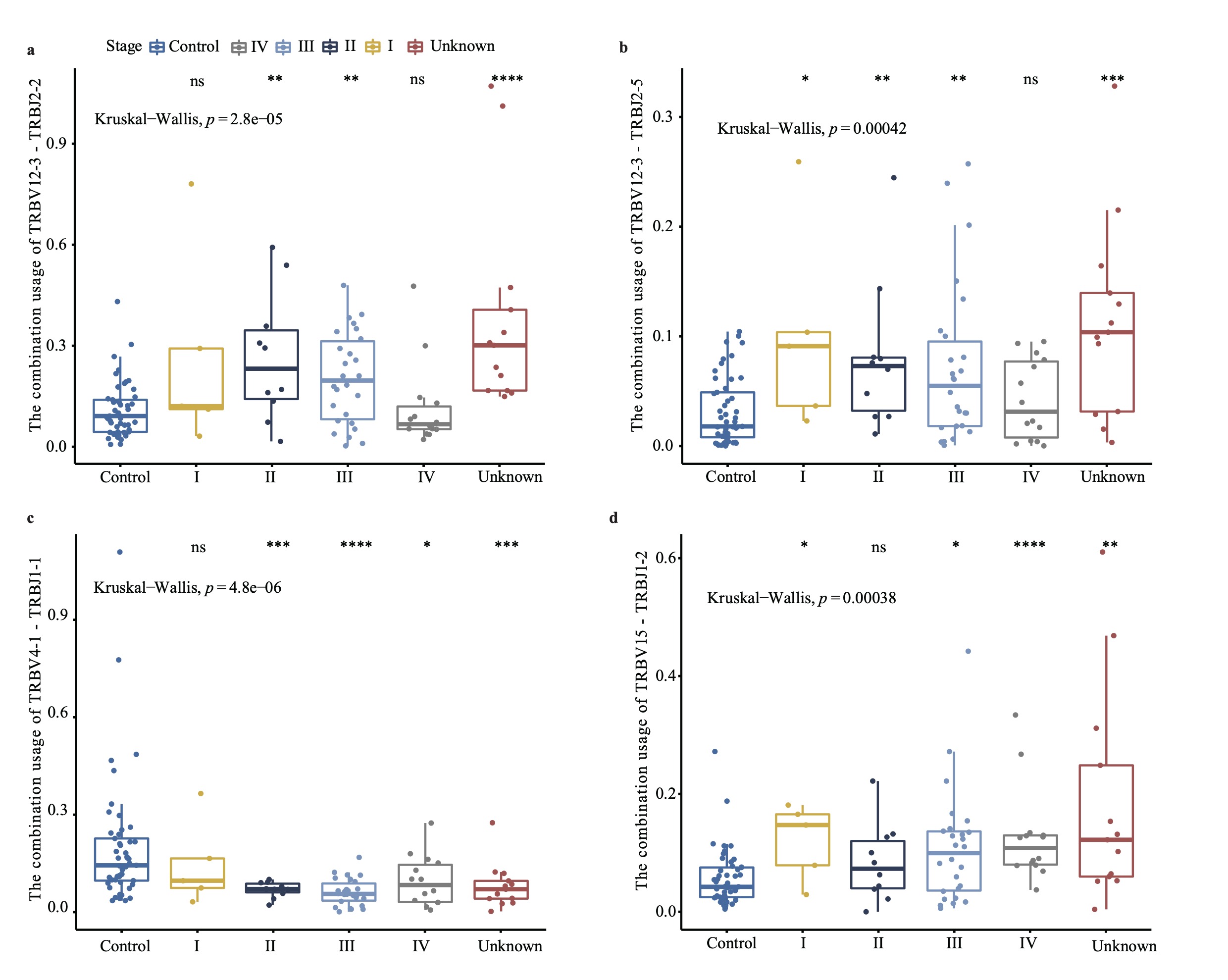

Supplement: Supplementary Figure 4 — Comparison of TRBV12.3-TRBJ2.2 (A), TRBV12.3-TRBJ2.5 (B), TRBV4.1-TRBJ1.1 (C) and TRBV15-TRBJ1.2 (D) combination usage between the control group and sub-tumor group of different tumor stage (Kruskal-Wallis test). [file Image_4.jpeg]

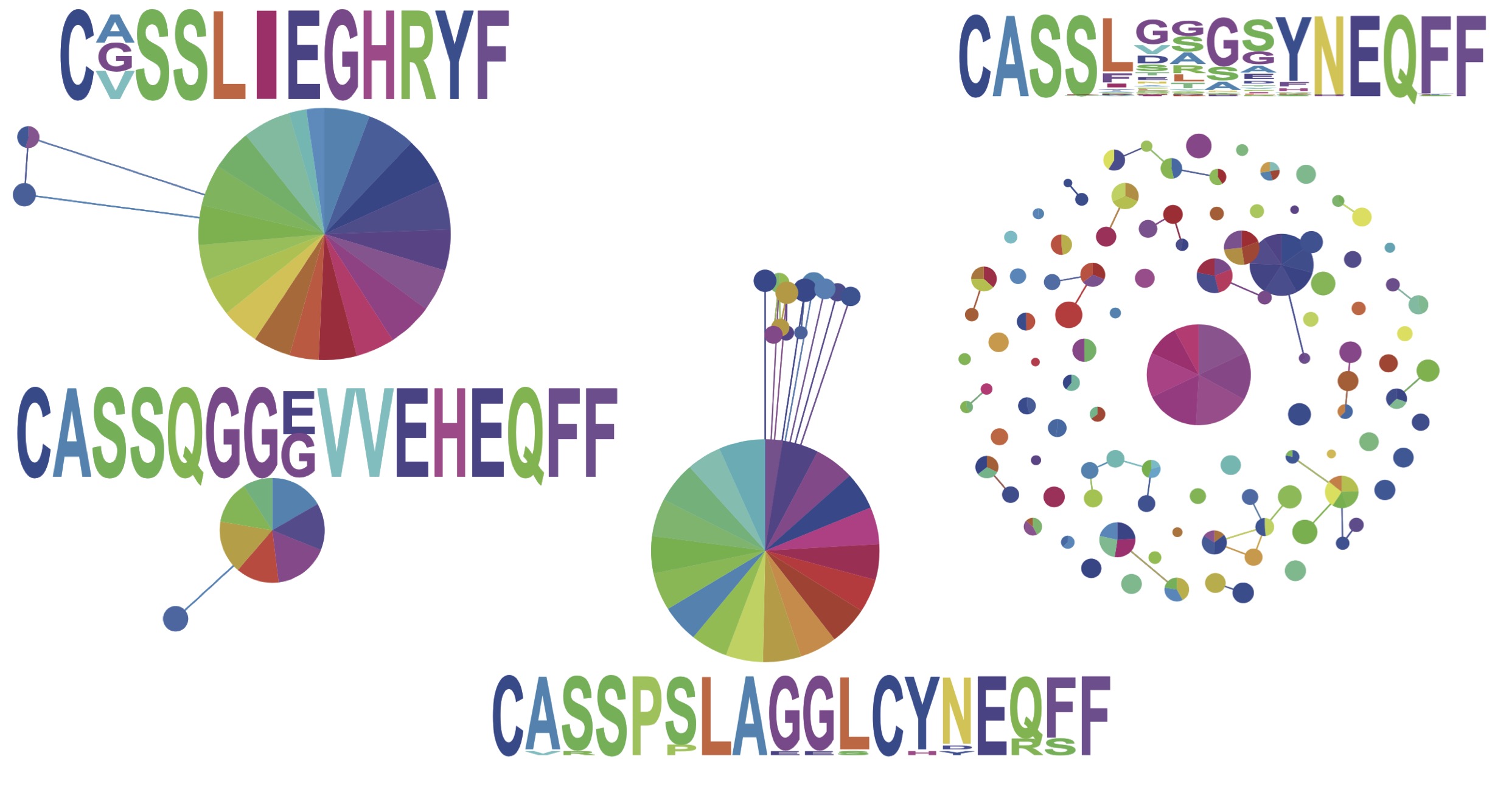

Supplement: Supplementary Figure 5 — The clusters plot of the top 4 hyper-expanded tumor specific TCR CDR3. Each cycle denotes a CDR3, the size of the cycle is in proportion to the frequency of the CDR3. The CDR3 shared by multiple sample are exhibited in pie chart with multiple colors. The linked CDR3 differ in just have one amino acid. The sequence logo of each CDR3 clusters are added to the cluster plot to reflect CDR3 sequence conservation. [file Image_5.jpeg]
